# Randomized Controlled Trial of Home Telemonitoring of Blood Pressure with an Adapted Tensiometer with SMS Capability

**DOI:** 10.3390/ejihpe13020033

**Published:** 2023-02-12

**Authors:** Renzo Calderón-Anyosa, Jean Pierre Tincopa, Mabel Raza, Cesar P. Cárcamo

**Affiliations:** 1 Facultad de Salud Pública y Administración, Universidad Peruana Cayetano Heredia, Lima 15102, Peru; 2 Digital Transformation Research Center, Universidad Norbert Wiener, Lima 15046, Peru; 3 Facultad de Ciencias y Filosofía, Universidad Peruana Cayetano Heredia, Lima 15102, Peru

**Keywords:** telemedicine, hypertension, primary care, short message system, monitoring

## Abstract

Despite being a public health problem, less than a third of hypertensive patients manage to control blood pressure (BP). In this paper, we conducted a two-arm randomized controlled trial to investigate the efficacy of an SMS-based home BP telemonitoring system compared to usual care in patients with uncontrolled hypertension from a primary care center. This study was conducted between April and August 2018. Participants in the intervention arm used a custom-designed telemonitoring device for two weeks and were followed up for two additional weeks; controls were followed for 4 weeks. The main objective of this study is to evaluate the impact on blood pressure of a telemonitoring system using a blood pressure monitor adapted to send data via SMS to health providers in primary care centers for 4 weeks. In this trial, 38 patients were included in the analysis (18 in each arm), 68% were women, and the mean age was 68.1 [SD: 10.8 years], with no differences between arms. Among the results we found was that There was no significant difference in the change in systolic BP values between the control and intervention arm (−7.2 [14.9] mmHg vs. −16.3 [16.7] mmHg; *p* = 0.09). However, we found a significant difference in the change of diastolic BP (−1.2 [6.4] mmHg vs. −7.2 [9.8] mmHg; for the control and intervention arms, respectively *p* = 0.03). With all this, we conclude that an SMS-based home BP telemonitoring system is effective in reducing diastolic BP by working in conjunction with primary care centers. Our findings represent one of the first interventions of this type in our environment, being an important alternative for the control of high blood pressure.

## 1. Introduction

High blood pressure is a public health problem, resulting in 15 million deaths per year worldwide [1]. The prevalence in Latin America varies from 30 to 50%; only 23% of men and 35% of women with diagnosed hypertension have their blood pressure under control [2,3].

In Peru, it is estimated that only 39.3% of those with hypertension are under treatment, and only 20% have their blood pressure under control [4], but in rural areas, this value can be as low as 4.9% [5]. Due to the low level of control of hypertensive patients, several studies have proposed telemonitoring measures of blood pressure at home, which has managed to increase the proportion of controlled patients. Current hypertension clinical guidelines suggest the use of telemonitoring both for the diagnosis and for the treatment of high blood pressure [6,7].

Home blood pressure telemonitoring is defined as the process by which blood pressure readings at home are transmitted to a central health information center or electronic medical record for use by healthcare providers and patients [8]. There is a large variety of telemonitoring systems, with differences in measurement methods and communication systems, including data transmission via Bluetooth, Wi-Fi, and telephone lines, among others. The use of these devices may be limited in some contexts when they rely on external devices such as smartphones with Bluetooth technology or Wi-Fi hot spots at home [9], emphasizing the need for simple, cost-effective systems that are easy to use and acceptable to both patients and providers.

An alternative for the implementation of telemonitoring at home is the adaptation of blood pressure monitors for home use, enabling them to send data through text messages (SMS), a technology with wide penetration [10]. Some studies have shown the feasibility of adapting blood pressure monitors to send blood pressure data via SMS [11,12]. Despite the technological advantages of using this telecommunication system, there is little information regarding its clinical impact. The main objective of this study is to evaluate the impact on blood pressure of a telemonitoring system using a blood pressure monitor adapted to send data via SMS to health providers in primary care centers for 4 weeks.

## 2. Materials and Methods

### 2.1. Study Design

A randomized controlled trial was performed. The intervention arm received the telemonitoring system, while the control arm continued with the usual management. The main outcome was the difference in systolic blood pressure (SBP) and diastolic blood pressure (DBP) at follow-up. The blood pressure monitors used in this study were adapted to send SMS using the services of a local mobile telecommunications provider.

The data were obtained confidentially. Personal identifiers were stored separately in a password-protected database to which only the researchers had access. The study protocol was registered on clinicaltrials.gov (NCT03524456).

### 2.2. Participants’ Enrollment

This study included patients with uncontrolled high blood pressure treated at the Condevilla health center in the district of San Martín de Porres, in the province of Lima. Patients with uncontrolled treated hypertension were defined as those with SBP above 130 mmHg or DBP above 80 mmHg [8].

The inclusion criteria were the following: (i) Patients older than 18 years; (ii) Diagnosis of high blood pressure for at least 3 months; (iii) Uncontrolled blood pressure; and (iv) Under treatment with antihypertensive medication. Exclusion criteria were (i) Patients on hemodialysis or peritoneal dialysis for chronic kidney disease; (ii) Pregnant women, and (iii) Patients planning to travel or change of address within a month of enrollment.

### 2.3. Randomized Grouping

Patients were referred to the study by the Condevilla health center. If they met the inclusion and exclusion criteria, they were invited to participate in the study and asked to provide informed consent. Randomization was performed in complete blocks of size 4.

### 2.4. Sample Size

For a significance level of 95% and 80% power, a standard deviation of 10 and 12 mmHg for the control and intervention arm and a sample size of 20 participants per arm would be required to detect differences of at least 10 mmHg [13].

### 2.5. Intervention

#### 2.5.1. Development of the Telemonitoring System

**Figure 1:**
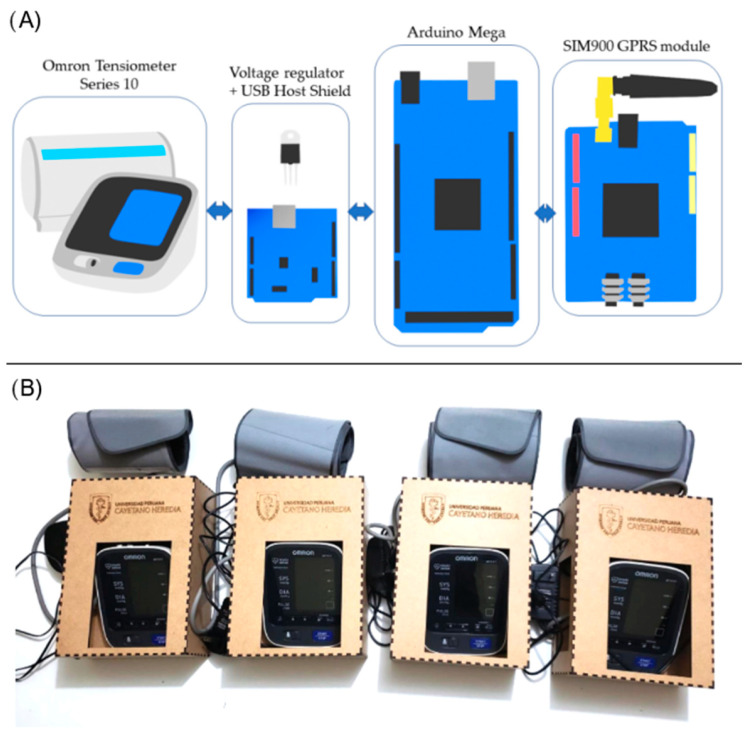
Total assembly of the telemonitoring equipment. (**A**) Connections between components (**B**) Final version with MDF casings.

A commercial Omron Series 10^®^ blood pressure monitor was used, which has USB (universal serial bus) connectivity from the factory. The USB port built into the blood pressure monitor was used to link it to the data capture and delivery module (Figure 1). The monitor itself was not modified. The cost of this equipment was around 70 USD. The accuracy of this monitor is recognized by the AHA (American Heart Association), and it has been used in other studies [14]. The equipment is easy to use and was designed for home use, with an easy-to-set cuff and a large number display. It shows the values of systolic and diastolic blood pressure, as well as heart rate, on an LCD screen.

#### 2.5.2. Hardware Development

The data capture and delivery module used Arduino development boards. Arduino is an open-source hardware and software company that designs and manufactures single-board microcontrollers and microcontroller kits for building digital devices and interactive objects. Arduino boards are commercially available, with a wide catalog of devices and kits [15].

#### 2.5.3. Software Development

Arduino has its integrated development environment (Arduino Integrated Development Environment IDE. It is a cross-platform application (for Windows, macOS, and Linux). This platform allows users to develop, compile and upload programs to an Arduino board.

#### 2.5.4. Telemonitoring Protocol

Participants in the intervention arm received the telemonitoring equipment and were asked to take at least two blood pressure measurements in the mornings and two in the evenings for two weeks. The blood pressure measurements were sent to the health personnel at the Condevilla health center, who made clinical decisions based on the measurements. These decisions could be adjustments or changes in medication or calls to the patient for appointments at the center.

Participants in the control arm received the status quo, consisting of monitoring based on visits to the health center for blood pressure determinations and treatment indications.

#### 2.5.5. Technical and Process Aspects

The technical and process aspects of home blood pressure monitoring are important to ensure accurate and reliable readings and to allow for effective monitoring and management of the patient’s blood pressure.

- Patient training: The patient is trained on how to properly use the blood pressure monitor and how to accurately record the readings.
- Equipment: A blood pressure monitor with telemonitoring capability, such as an adapted tensiometer with SMS capability, is provided to the patient.
- Monitoring frequency: The frequency of monitoring was determined and agreed upon between the patient and healthcare provider in two measurements in the morning and two measurements at night spaced out by 5 min each daily, after 1 min of rest.
- Data transmission: The patient takes their blood pressure readings, and the telemonitoring capability of the blood pressure monitor transmits the data to their healthcare provider using via SMS.
- Review by a healthcare provider: The healthcare provider receives the transmitted data and reviews the readings to monitor the patient’s blood pressure and identify any potential issues.
- Follow-up: The healthcare provider may schedule a follow-up appointment with the patient, if necessary, based on the review of the transmitted data.

### 2.6. Study Setting

Outpatients of the health center were invited to participate in the study after verifying the inclusion and exclusion criteria. The patients were informed of the study objectives, procedures, and requirements. Consenting patients were asked to sign the informed consent form.

After randomization, those in the intervention arm were given a training session in the use of the telemonitoring system. They were provided with information on the proper placement of the cuff, as well as on the correct position of the body during blood pressure measurement. They were given an informative guide as well as a user manual.

Study personnel delivered the monitors to participants during home visits. The most suitable place for taking blood pressure was selected to suit the patient. After choosing the place, the signal of the equipment and the reception of the message were both verified through a blood pressure measurement (not considered in the analysis).

**Figure 2:**
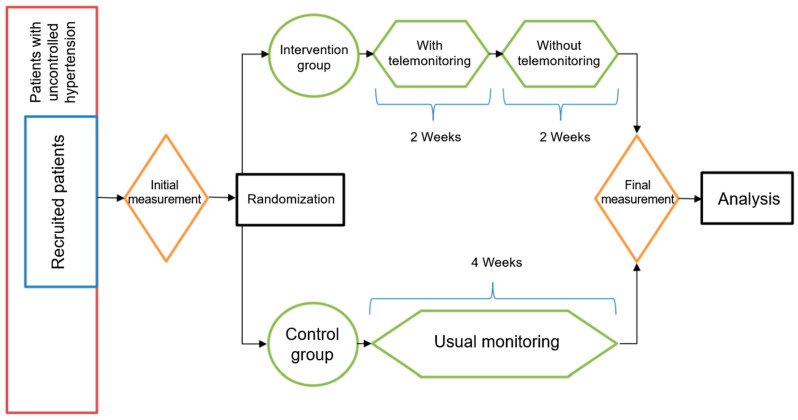
Design of the intervention.

Participants skipping more than two consecutive blood pressure measurements were contacted by telephone to evaluate the reasons for the discontinuation and to encourage continuous monitoring. The decision to adjust the medication or call the patient was given by the health center’s physician. The final measurement was taken two weeks after completing the telemonitoring. For participants in the control arm, baseline and follow-up measurements were taken four weeks apart (Figure 2).

### 2.7. Statistical Analysis

The primary statistical analysis was performed using the Student’s *t*-test for two independent samples comparing the mean change in blood pressure between intervention and control arms. The change in blood pressure was the difference between the four-week value and the baseline value. Qualitative variables have been expressed as a percentage. A Fisher’s exact test, or a Chi^2^ test, was used for the comparison of proportions. For normally distributed quantitative variables, means and ±standard deviations are presented. A comparison of means was performed using the Student’s *t*-test. Non-normally distributed quantitative variables were described with medians and interquartile ranges, and comparisons were performed using the Mann–Whitney test. Associations with *p* values < 0.05 were considered statistically significant. Statistical analysis was performed using the STATA 15 (StataCorp, College Station, TX, USA).

## 3. Results

### 3.1. Recruitment of Participants

**Figure 3:**
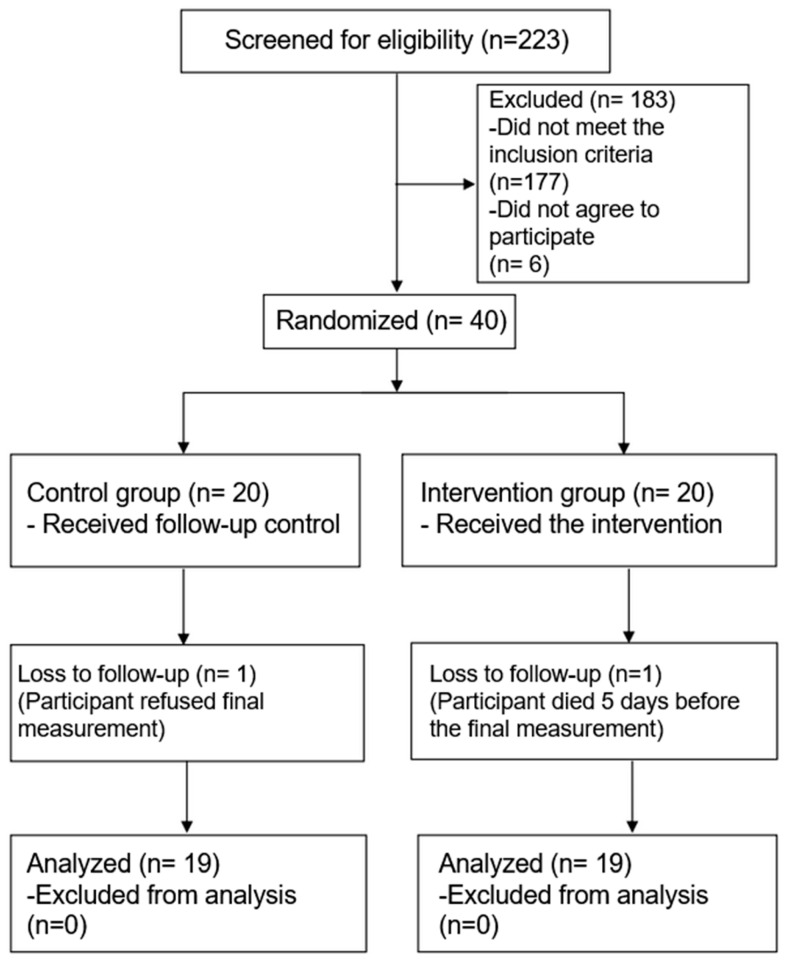
CONSORT flowchart of participating individuals throughout the essay.

The recruitment and follow-up of patients took place between April and August 2018. A total of 223 patients were evaluated for eligibility, of which 183 patients were excluded (134 did not present a diagnosis of arterial hypertension, 23 had less than 3 months of diagnosis, 20 were not on pharmacological at screening, and 6 patients did not agree to participate in the study). A total of 40 patients were randomized: 20 to the control arm and 20 to the intervention arm. One of the patients in the control arm withdrew before the second blood pressure measurement. Likewise, one patient in the intervention arm died days before the second blood pressure measurement was performed; thus, 38 participants were included in the final analysis, 19 in each arm (Figure 3).

### 3.2. Baseline Characteristics

#### 3.2.1. Demographics Data

The majority of participants (26; 68.4%) were female. The mean age of 68.1 ± 10.8 years, and the mean BMI was 29.5 ± 4.0 kg/m^2^. 14 (36.8%) of the participants were born in Lima), 22 (57.9%) were married or cohabiting, 24 (63.2%) had only primary education, and 20 (52.6%) were housewives. The only significant difference found within the demographic and clinical variables between the two study arms was marital status (*p* = 0.026).

#### 3.2.2. Clinical Data

The mean time since diagnosis of high blood pressure was 11.1 ± 7.7 months, while the most frequent pharmacological treatment was angiotensin-converting enzyme inhibitors (ACEI) (*n* = 24; 63.2%). Likewise, it was found that the majority of participants had a family history of hypertension (*n* = 21; 55.3%). The most common comorbidity was Diabetes Mellitus *(n* = 9; 23.7%). No differences were found in clinical variables between the two study arms (*p* > 0.05) (Table 1).

In the total sample, the mean SBP at the baseline measurement was 156.7 ± 13.7 mmHg, while the DBP was 86.6 ± 9.6 mmHg; there were no differences in blood pressure between control and intervention arms at baseline. Regarding the final measurement, the mean SBP was 144.9 ± 15.8 mmHg, and the DBP was 82.3 ± 10.6 mmHg; no differences between study arms were found (Table 2).

### 3.3. Primary Outcome

The difference in SBP values (final measurement—initial measurement) was −16.3 ± 16.7 mmHg in the intervention arm, while in the control arm, it was −7.2 ± 14.9 mmHg (*p* = 0.087). Similarly, the difference in DBP values was −7.2 ± 9.8 mmHg in the intervention arm, while in the control arm, it was −1.2 ± 6.4 mmHg (*p* = 0.032).

## 4. Discussion

Our study shows a reduction in SBP values and a significant reduction in DBP values in those participants who used the telemonitoring system compared to the control arm. These results show the potential benefit of including telemonitoring systems in primary care centers for the control of hypertensive patients, and if scaled up, these systems represent an important alternative strategy for public health.

This study shows that immediately after using the telemonitoring system, the reduction in blood pressure values is greater than after discontinuing its use for two weeks. This could be because the effect is lost after the equipment use stops since the effect could be related to the measurement, visualization of results, and communication with the doctor. 

The reduction in SBP and DBP values found in our study is consistent with that reported in previous studies. A meta-analysis that evaluated clinical trials involving telemonitoring systems for blood pressure control found an average reduction in SBP of −4.8 mmHg and DBP of −2.1 mmHg, with some studies showing reduction values as large as −25 mmHg and −15 mmHg for SBP and DBP, respectively [16]. Our results show an average reduction in SBP of −16.3 ± 16.7 and DBP of −7.2 ± 9.8. This greater reduction could be because the baseline blood pressure values were higher in our population compared to most of the studies included in this meta-analysis.

A study conducted in the United States, which evaluated an intervention with a blood pressure telemonitoring system with medication adjustment by pharmacists, found after 6 months of follow-up, mean reductions of −6.0 (SBP) and −2.0 mmHg (DBP), significantly higher than those in controls [17]. Although the follow-up time was shorter in our study, suggesting that blood pressure goals can be achieved in a shorter follow-up time.

On the other hand, our results are comparable with studies that used more sophisticated telecommunication systems. A study conducted in Germany that evaluated a telemonitoring system using a blood pressure monitor connected to a mobile phone via Bluetooth found mean reductions in the intervention arm of −17.0 (SBP) and −9.0 mmHg DBP [18] significantly larger than those in the control arm. It should be noted that our sample consisted mainly of patients older than 70 years, while this study had a mean age of 55 years.

Age can represent a limiting factor for the use of some telecommunication systems, resulting in a barrier to the implementation of telemonitoring systems [8]. The simplification of processes and the elimination of the need for additional equipment represents an advantage of our study, allowing access to patients unfamiliar with modern technology.

Regarding the use of telemonitoring systems in primary care centers, information is scarce [19], and there are mainly studies that have evaluated their acceptability and feasibility [20,21] without presenting or evaluating their clinical effect. A study conducted in the United Kingdom, which evaluated a telemonitoring system at the first level of care, found reductions in SBP and DBP after 6 months of follow-up similar to those found in our study, showing that care at primary centers can be improved by the use of telemonitoring systems [22].

Regarding the causal relationship of the effectiveness of the telemonitoring system, we speculate that in our study, it could be due to an improvement in adherence to treatment, but we have the limitation of not having evaluated this parameter in this study.

## 5. Conclusions

Our study shows a reduction in SBP values and a significant reduction in DBP values in those participants who used the telemonitoring system compared to the control group. These results show the potential benefit of including telemonitoring systems in primary care centers for the control of hypertensive patients, and if scaled up, they represent an important alternative strategy for public health.

The telemonitoring system helps improve adherence to treatment, also improving disease awareness. When patients see the blood pressure values, they have an objective parameter to base themselves on; likewise, knowing that there is someone on the other side continuously reviewing the values helps them to continue and not stop taking the medication.

Part of the future work that could be generated from this study is to take it to rural environments where the only constant telecommunication signal is SMS, in addition to evaluating this tool for longer follow-up times and with a larger sample size.

## Figures and Tables

**Table 1:** General characteristics of the participants by study arms.

	Control	Intervention	Total	*p*
**Female Sex**	16 (84.2%)	10 (52.6%)	26 (68.4%)	0.079
**Age (years)**	68.7 ± 11.5	67.5 ± 10.3	68.1 ± 10.8	0.744
**BMI (kg/m^2^)**	29.4 ± 3.4	29.5 ± 4.6	29.5 ± 4.0	0.988
**Born in Lima**	5 (26.3%)	9 (47.4%)	14 (36.8%)	0.313
**Marital status**				
Single	3 (15.8%)	2 (10.5%)	5 (13.2%)	0.026
Married/Cohabiting	7 (36.8%)	15 (79.0%)	22 (57.9%)	
Divorced	1 (5.3%)	0	1 (2.6%)	
Widow/widower	8 (42.1%)	2 (10.5%)	10 (26.3%)	
**Education level**				
Primary	13 (68.4%)	11 (57.9%)	24 (63.2%)	0.788
High school	5 (26.3%)	6 (31.6%)	11 (29.0%)	
Superior	1 (3.3%)	2 (10.5%)	3 (7.9%)	
**Occupation**				
Housewife	11 (57.9%)	9 (47.4%)	20 (52.6%)	0.455
Casual work	2 (10.5%)	5 (26.3%)	7 (18.4%)	
Permanent job	6 (31.6%)	5 (26.3%)	11 (29.0%)	
**HTA diagnosis time (months)**	10 (5–12)	10 (3–16)	10 (5–15)	0.918
**Treatment**				
ACEI	15 (79.0%)	9 (47.4%)	24 (63.2%)	0.248
ARA-II	3 (15.8%)	5 (26.3%)	8 (21.1%)	
CCB	0	1 (5.3%)	1 (2.6%)	
ARA-II + CCB	1 (5.3%)	3 (15.8%)	4 (10.5%)	
ACEI + CCB + ARA-II	0	1 (5.3%)	1 (2.6%)	
**Family history of high blood pressure**	10 (52.6%)	11 (57.9%)	21 (55.3%)	1
**Comorbidities**				
Diabetes	6 (31.6%)	3 (15.8%)	9 (23.7%)	0.447
Others	14 (73.7%)	12 (63.2%)	26 (68.4%)	0.728

HTA: Arterial hypertension, ACEI: Angiotensin-converting enzyme inhibitors, ARA-II: angiotensin II receptor antagonists, CCB: calcium channel blockers.

**Table 2:** Change in blood pressure values between the baseline measurement and the final measurement of the study arm.

	Control	Intervention	Total	*p*	P MW
**Baseline measurement (BM)**					
SBP (mmHg)	157.0 ± 15.2	156.3 ± 12.4	156.7 ± 13.7	0.880	0.9301
DBP (mmHg)	84.8 ± 9.1	88.3 ± 9.9	86.6 ± 9.6	0.276	0.3128
**Final measurement (FM)**					
SPB (mmHg)	149.8 ± 17.4	140.1 ± 12.6	144.9 ± 15.8	0.056	0.111
DBP (mmHg)	83.6 ± 10.9	81.1 ± 10.3	82.3 ± 10.3	0.460	0.6180
**FM–BM**					
SBP (mmHg)	−7.2 ± 14.9	−16.3 ± 16.7	−11.7 ± 16.3	0.087	0.0470
DBP (mmHg)	−1.2 ± 6.4	−7.2 ± 9.8	−4.2 ± 8.7	0.032	0.0452

SBP: systolic blood pressure, DBP: diastolic blood pressure.

## Data Availability

The data that support the findings of this study are available from the corresponding author upon reasonable request.
